# Impacts of Incentive and Disincentive Mechanisms for Ensuring Environmentally Friendly Livestock Waste Management

**DOI:** 10.3390/ani12162121

**Published:** 2022-08-19

**Authors:** Deng Yue, Apurbo Sarkar, Chen Guang

**Affiliations:** 1 School of Management, Xi’an University of Science and Technology, Xi’an 710054, China; 2 College of Economics and Management, Northwest A&F University, 3 Taicheng Road, Xianyang 712100, China

**Keywords:** environmentally friendly technology, waste management, manure, carbon emissions, technological progress, reduction, structural equation modeling (SEM), China

## Abstract

**Simple Summary:**

This study evaluated the impacts of incentive and disincentive mechanisms in ensuring environmentally friendly livestock waste management, drawing on a survey of 499 pig farmers from China. An assessment framework was developed and empirically tested using partial least squares structural equation modeling. The findings suggest incentives and disincentives can be effective in ensuring adoption of environmentally friendly livestock waste management practices. However, the disincentive mechanism demonstrated relatively lower interaction values than the incentive mechanism. The survey results indicated that discounts, subsidies, and training provision were the most impactful factors. In summary, the balance and interactions between incentives and disincentives need to be well-planned and managed to strengthen farmers’ adoption of environmentally friendly livestock waste management practices.

**Abstract:**

Environmentally friendly waste management (EFWM) is a safer way of waste disposal that can foster a cleaner environment for both farms and their surroundings. It may lessen land, air, and water pollution, as well as moderate ecological footprints, and aid in sustainable agricultural development, which has become one of the major concerns of the modern era. To achieve these outcomes, incentives and control mechanisms initiated by the government may alter farmers’ behavior. The study involved a review of relevant literature and the conduct of interviews with 499 pig breeders to evaluate the impacts of government incentives and control mechanisms on fostering the adoption of environmentally friendly waste management practices by farmers. A theoretical framework based on existing studies is proposed, utilizing a structural equation modeling (SEM) approach to analyze the data and illustrate the relationships among incentives and control mechanisms. The results show that: (i) overall the impacts of incentive mechanisms were stronger and more effective than those of control mechanisms. Among them, subsidy policy and discount policy were the most influential for farmers’ adoption behavior. However, penalty and disincentive policy also impacted the outcome variables; (ii) a significant relationship was observed among regulatory, disincentive, and subsidy policies and a moderate relationship among penalty, insurance, and discount policies. However, bonus-community service and social critic policies did not show any significant relationship with any other variables. The research findings can assist the Chinese government in gaining a comprehensive understanding of the impacts of two crucial mechanisms and promoting the adoption of environmentally friendly practices by farmers. The government should highlight and strengthen the importance of social obligations and orientation, as well as providing monetary support at the rural level to improve farmers’ ability to adapt to environmentally friendly waste management practices.

## 1. Introduction

In 2016, the Chinese government signed the “Paris Agreement”, promising to reduce carbon emissions per unit of GDP by 60–65% in 2030 compared to 2005 [1]. Along with all the other signatories to the Paris Agreement in 2015, China agreed to make changes to try to keep global warming at 1.5^C^ above pre-industrial levels, and “well below” 2^C^ [2,3]. Compared with the objectives set in 2016, the new targets are more ambitious in the timeframe. In an address to the 75th session of the UN General Assembly (UNGA 75), President Xi Jinping declared that China will seek to ensure that CO_2_ emissions peak before 2030 and that carbon neutrality is achieved before 2060 [4]. At the beginning of 2021, the Chinese government proposed the “13th Five-Year Plan” to emphasize green development, with a focus on improving the ecological environment, and moving to green development methods that utilize natural resources in a planned way [5], minimizing environmental damage [6]. The Plan also focuses on improving the ecological environment and highlights the importance of reducing waste across various sectors [7]. The report of the Fifth Plenum of the 19th Communist Party Congress of China stated the importance of environmental friendliness as a core long-term goal for socialist modernization [8]. Agricultural development and livestock farming are expected to contribute to the reduction in environmental impacts, since pollution produced by this sector alone would exceed pollution targets in the context of rising temperatures. The production, transportation, and consumption of food are very carbon-intensive, and utilize vast amounts of fossil fuels that generate pollution in the atmosphere, accelerating global warming [6].

The livestock industry is an important component of the planetary food chain that helps to alleviate poverty, maintain food and nutrition security, and promote economic growth. According to the FAO, livestock contributes 40% of the global value of agricultural output and supports the livelihoods and food and nutrition security of almost 1.3 billion people [9,10]. Livestock plays a primary role in the development of sustainable food systems; for example, manure is an important source of natural fertilizer, and livestock utilized as draft animals can enhance production in areas with minimal automation, and represents a crucial asset for vulnerable communities. Locally, animal production techniques can help to preserve biodiversity and carbon absorption in land and biomass. In harsh environments, such as highlands and drought-prone areas, livestock is frequently used as the only means of sustainably converting natural resources into food, fiber, and labor power for local populations. Increasing demand and changing consumption trends have occurred as a result of rising earnings, changing dietary habits, and exponential population expansion, making the livestock industry one of the most rapidly expanding agricultural sub-sectors in middle- and low-income nations. Throughout the livestock production system, this creates significant opportunities for smallholder farmers, businesses, and entrepreneurs. However, if not properly managed, this growth risks aggravating sustainability issues that span issues of equity, environmental impact, and public health. The existing literature (such as Borlée et al. [11], Sorathiya et al. [12], and Sahoo et al. [13]) highlights that the livestock sector is one of the most strongly polluting sectors within the agricultural industry, with livestock farming accounting for 10–12% of the global emissions of carbon dioxide equivalents (CO_2_e) [14]. Livestock are responsible for around 64% of total ammonia discharges, resulting in significant acid rain and acidity in the environment, and is also responsible for high levels of methane production, contributing 35–40% of global methane emissions [15]. Alarmingly, animal waste pollution has caused highly publicized and catastrophic waste spills that have contaminated large areas of surface and groundwater. Poor waste management facilities and farmers’ low awareness levels have worsened the situation. At the same time, there is significant scope to improve livestock waste management practices so that they are more sustainable, more equitable, and pose less risk to animal and human health. The situation described highlights the importance of environmentally friendly waste management practices.

Environmentally friendly waste management simply means the collection, transport, processing or disposal, managing, and monitoring of waste materials to minimize their impacts on humans and the environment. Various studies have demonstrated that eco-friendly waste management can lessen carbon release and environmental degradation and ammonia pollution. For example, Shen et al. [16] illustrated an environmentally friendly animal waste disposal process for recovering ammonia and avoiding release into the atmosphere. In a study of 15 Russian pig farms, Izmaylov et al. [17] found that improved modern science and technological solutions enabled highly effective means of waste management which significantly lowered water resource depletion. In a critical review of livestock manure processing technology, Khoshnevisan et al. [18] highlighted the importance of EFWM in terms of energy production and lowering overall ecological impacts. Though existing studies have highlighted the importance of EFWM in terms of eco-protection, there is a lack of awareness among grassroots farmers concerning environmental pollution by the livestock industry. According to Koul et al. [19], farmers in developing countries are mostly apathetic toward environmental threats raised by their farm waste. Therefore, administrative interventions in terms of incentives and control mechanisms are widely used by governments worldwide. In a study of the Brazilian smallholder pig industry, Vilas-Boas et al. [20] found that strict governmental policies, such as preventive and control policies, significantly altered farmers’ production behaviors. Oenema [21] found that governmental interventions led to significant reductions in uncontrol animal manure management in the European Union. He recommended that government should enforce strict environmental legislation, combined with increasing public awareness of food safety, animal welfare, and landscape maintenance to trigger market spillover effects which would eventually help to ensure environmental and food safety. In the case of livestock manure management in Longyou, China, Xu et al. [22] found that monetary and other forms of subsidies significantly impacted farmers’ adoption of environmentally responsible manure management tactics. 

There are some valuable studies which have considered the stimulating effects of governmental regulations and support policies on environmentally friendly waste management within livestock industries [23,24,25]. However, the relationships between government incentives and control mechanisms have not yet been comprehensively evaluated. Moreover, most available studies have evaluated these two mechanisms separately [26,27]. To the best of our knowledge, this study is the first that considers incentives and control mechanisms within an integrated framework and evaluates these in the light of empirical evidence. The following research questions arise: (i) What are the structural relationships between governmental disincentive and incentive mechanisms in terms of environmentally friendly livestock waste management? (ii) Are incentive and disincentive mechanisms interdependent? (iii) How can incentives and disincentives be evaluated within an integrated framework? The investigation of these issues will help to provide clear and specific recommendations for decision-makers, which is the prime motivation of the research. The study involved the evaluation of the impacts of incentives and disincentive mechanisms in terms of ensuring environmentally friendly livestock waste management by employing a structural equation modeling approach. More specifically, the study proposes an integrated framework incorporating incentive and disincentive mechanisms and empirically tested the framework by employing partial least squares structural equation modeling (PLS-SEM) using the survey data of 499 pig breeders of China. The approach pursued has important theoretical and practical significance for ensuring adherence to environmentally friendly agricultural development and meeting China’s agro-emission reduction goals. The major contributions of the article are as follows: (i) We propose and test a structural framework of incentive and disincentive mechanisms and portray the interaction between these two; (ii) For policymakers, the study provides policy recommendations relevant to the local context and in a targeted manner, which will assist policymakers in the formulation of specific policies for specific regions. Table 1 represents all the variables and their descriptions. 

## 2. Theoretical Foundation and Hypothesis Development

Farmer’s willingness to adopt new practices and their adoption behavior are multidimensional concepts often quantified by assessment of farmers’ attitudes and norms [28]. While, according to the theory of reasoned action, there could be several possible factors influencing any individual’s attitudes and norms, some may motivate them and some deter them from any particular action (for more details, see Ajzen and Fishbein [29]). However, some studies, for example Knockaert et al. [30] and Zhang et al. [31], suggest that these mechanisms may impact farmer’s attitudes and subjective norms as mediators. Interestingly, among the various studies of the Chinese livestock industry, most researchers (e.g., Ge et al. [32], Si et al. [33], and Wang and Tao [34]) have observed that enabling an environmentally friendly waste management revolution is likely to be primarily influenced by governmental disincentive and incentive mechanisms. The present study adopts the variables and core model presented by Ge et al. [32] and modifies these in relation to the context of the study. In the following section, we outline the theoretical perspective and major components of the framework.

First, a rise in living conditions and dietary alterations has contributed to the rapid expansion of the global livestock industry, which has caused significant environmental harm owing to the improper management of animal waste [35]. Thus, governments have often adopted strict policies and provided significant policy supports, popularly known as incentive and disincentive mechanisms [32]. In the current livestock market conditions, the use of incentive-based policies is becoming more widespread worldwide because economic instruments offer a more flexible and cost-effective form of regulation than conventional measures [36]. Governments seek to influence farmers’ environmental preferences through subsidies and publicity, which play an important role in the treatment of livestock and poultry pollution [37]. In behavioral science, incentive mechanisms are a widely used tool to encourage individuals. In agricultural economics, an incentive can be in the form of either monetary or cognitive support [38]. There has been extensive investigation in the literature of the impacts of governmental incentive mechanisms on fostering positive behavioral change (e.g., Ge et al. [32], Adelodun et al. [39], and Siegford et al. [40]). 

In a study of the Brazilian livestock industry, Mathias [41] found that farmers are more likely to adopt sustainable waste management practices if the government provides favorable support. After reviewing relevant literature, Reza and Chen [42] suggested that government can play a pivotal role in enabling the transfer of technology and provision of technical support to positively influence the selection of appropriate waste management methods by farmers. With respect to incentive mechanisms, previous investigations have largely evaluated variables including: whether there are subsidies or not [43]; whether subsidies for technology and machines and materials improve the adoption of environmentally safe livestock manure management or not [44]; whether or not ecological quality incentive schemes are applicable [45]; whether linking insurance policy to incentives has any impact [46]; whether community service bonuses have any impacts on farmers’ behavior regarding environmentally friendly manure management [32]; whether governments provide technical training or not [26,47]; and what the impacts of loan interest discount policy might be [48]. 

In an in-depth analysis of 496 pig farmers in three provinces of China, Si et al. [33], found that that government subsidies significantly influenced farmers’ behavior regarding safer waste disposal behavior. Zhang et al. [49] found that specific and targeted policy incentives and outreach could profoundly influence farmers to adopt improved recycling of animal manure, reducing negative environmental impacts directly, and indirectly through reduction in the use of synthetic chemical fertilizers. In a study of swine and cattle farmers in Brazil, Mathias [41] found that government incentives for the use of biodigesters significantly increased farmers’ willingness to choose effective waste treatment and management and turn waste into resources. Tao and Wang [50] explored the impacts of governmental reimbursement policies among livestock and poultry framers of Shangdong, China, and found that a significant number of farmers were influenced by government incentive policies towards utilizing waste as a resource. In an evolutionary game approach regarding the recycling of manure in the breeding industry in Hunan Province, Xiong et al. [51] found that local government incentives represented the best means of motivating farmers to use manure recycling methods. Karlsson et al. [52] examined Swedish farms’ agriculturally based biogas production and suggested that biogas production on farms may become feasible with the help of long-term government subsidies and other forms of incentive, not only in terms of societal and ecological benefits but also in terms of financial benefits. Based on the above discussion, hypothesis 1 is proposed:

**Hypothesis** **1** **(H1).** *The incentive mechanisms used by government may impact farmers’ adoption of environmentally friendly livestock waste management practices*.

However, disincentive mechanisms represent the conventional method of ecological preservation, whereby authorities specify procedures that individual polluters should follow, often coupling this with substantial penalties for breaches [53]. According to Wang et al. [54], if a livestock breeder risks a fine or social criticism for not managing their farm’s waste, they usually pay more attention, which can eventually increase the adoption of scientific technology and proper waste management standards. When it comes to regulating pollution, the government is primarily responsible for setting and enforcing ecological standards [55]. The Chinese government has proposed various policies and measures for environmental governance that have acted as disincentive mechanisms to promote the recycling of sick and dead pig waste. There are several potential positive and negative externalities associated with livestock farming which can trigger pollution if they are not treated effectively. The prevention of pollution spillovers requires the strict implementation of governmental environmental legislation that requires livestock breeding firms to reduce pollution and ensure effective means of waste management. Interestingly, with respect to disincentive mechanisms, previous studies have assessed variables such as whether relevant governmental bodies have monitored the farmers’ activities [56,57], whether monetary penalties encourage farmer behavioral change [58], whether the area has any corresponding regional penalties for not properly managing waste [59], and whether policies of cracking down on trading and restricting market access exert any significant influence on proper livestock waste management [32,60]. 

In a comprehensive study of sustainable livestock farming in the Jiuzhou River basin in China, Sun et al. [61] found that governmental regulation remains a key factor in determining whether farms will adopt circular economic systems and eventually adopt sustainable waste recycling methods. YuJun et al. [62] explored the status of harmless livestock waste treatment in China and found that the stricter the governmental policies, the better the behavior of farmers with respect to waste management. Centner et al. [63] advocated a disincentive framework that seeks to prevent growers from being involved in activities that contribute to spreading contamination. They also highlighted the importance of integrating disincentive mechanisms with local farmers’ rights and water body laws for preventing inappropriate manure management. In a study of Chinese pig farming, Wu et al. [64] found that government supports and penalty mechanisms were both crucial; however, in terms of shaping farmers’ behavioral intentions, governmental regulation and penalty policies were more effective than incentives. In a study of the Canadian livestock sector, Cleary et al. [65] found that government regulations and disincentives had a positive impact on farmers’ choice of effective waste management methods. Based on the above discussion, hypothesis 2 below is proposed:

**Hypothesis** **2** **(H2).** *Disincentive mechanisms pursued by the government have a positive impact on farmers’ behavior towards the adoption of environmentally friendly livestock waste management*.

## 3. Materials and Methods

### 3.1. Methods

The research strategy involved a combination of quantitative and qualitative methodologies. The variables, the items of the latent constructs, and the content of the questionnaire were adopted from the existing literature, adjusted and finally tested with an empirical dataset. Partial least squares-structural equation modeling (PLS-SEM) was utilized for determination of the relationships between variables. According to Lee et al. [66], PLS-SEM is one of the best approaches where the theoretical framework needs to be tested with empirical evidence. It is an estimation approach using a structural equation model that integrates an advanced statistical approach with the forecasting and simulation of latent variables, leading to enhanced conceptual modeling capability [67]. 

The main reason for choosing PLS-SEM was as follows [68,69]: (i) It can manage highly complicated concepts that include a large number of variables and structures; (ii) It is flexible and efficient when normalization cannot be assured and can deal with missing data; (iii) It can provide relatively comprehensive outcomes with a limited dataset; (iv) It can derive determinate scores for latent variables, which may then be used in future studies. As a result of its ability to operate with small datasets, its convergence with minimal theory, and its high prediction accuracy, PLS-based SEM has been regarded as preferable to other SEM strategies, including covariant base (CB-SEM), GSCA, and NEUSREL. Several studies using similar dimensions to represent farmers’ adoption behavior have used PLS-SEM (such as Wang et al. [70], Sarkar et al. [71], and Omar et al. [72]). In SEM, the indicators are usually aligned in two forms, reflective and formative, to determine the main framework of the measurement model. The indicators used in a reflective framework are driven by the fundamental conceptual framework and possess constructive and, preferably, strong correlations with each other [73]. In a formative model, the indicators do not always correspond to a single theme, and, as a result, there is no established sequence of association among components [74]. Consistent with the core framework of the study, as the indicators represent latent variables, we have chosen a reflective measurement model, as suggested by Hair et al. [75] and Wong [76]. Interestingly, farmers’ adoption of environmentally friendly waste management practices is a latent variable that cannot be observed using a single construct. Instead, it is explored using a set of indicators which are reflectively interconnected with the two fundamental latent variables (i.e., incentives and disincentives). Therefore, we used SmartPLS 3 (SmartPLS GmbH, www.smartpls.com (accessed on 12 June 2022)) software to perform the analysis as it is considered superior software to other relevant tools, such as AMOS and LISREL [76]. The exact procedures and stages in performing the PLS-SEM assessment were as follows: (i) The first phase involved the assessment of the measurement model. The assessment of the measuring model confirmed the constructs’ dependability, composite reliability, and discriminant validity; (ii) In the second step, the structural model was evaluated, and the level of significance of the path coefficients was determined to test the hypotheses; (iii) To calculate the standard deviations of the estimations, a bootstrapping method was used for 50,000 separate simulations.

### 3.2. Sample and Data Collection

The current work was carried out in the livestock industry in China and utilized a survey method to collect responses from Chinese pig breeders. A farmhouse was considered as the unit of analysis and only those farmers who were familiar with environmentally friendly livestock waste management were targeted. We carried out face-to-face interviews, along with administration of a survey questionnaire, to attain the research objectives. The questionnaire was pre-tested for its validity and readability before the collection of data. The questionnaire was discussed with industry experts and academics for pre-testing, and the suggested changes were made accordingly. A seven-point Likert scale was employed to record responses. A multilayered random sampling approach was used to select the 499 respondents. 

In a simple random sampling approach, selection is based on a simple probability where each of the potential samples is given an equal opportunity of being chosen [73]. In this stage, the investigators, led by the team leader, took part in discussion with relevant officers of the provincial animal husbandry bureau to gain a clear view of the region’s characteristics. From the eight counties, two or three towns were randomly chosen. From those townships, we selected two or three villages with a simple random sampling technique which provided us with 41 villages in total to perform the final survey. Finally, the study selected ten to fifteen pig breeders from each village using a simple random sampling technique, resulting in 587 pig breeders in total. Throughout the entire sampling process, it was ensured that only farmers with some prior knowledge/experience regarding environmentally friendly livestock waste management were selected. After eliminating incomplete data, the study finally used data for 499 pig breeders for further exploration and processing. Before starting each formal interview, the interviewer briefly discussed the terms and content of the questionnaire with the respondent, so that they were fully aware that they were able to refuse to answer any questions and could opt out at any time during the interview After obtaining respondent’s verbal permission and consent, the final interview was performed. The interviews and questionnaire did not seek to acquire personal information and were solely based on essential identifiers collected solely for analytical purposes. Therefore, there was no requirement to obtain prior permission from an internal or external ethical review board, as suggested by Tambotoh et al. [77] and Lindorff [78]. 

In any form of empirical analysis, the minimum sample size is a major concern. As a multidimensional approach, PLS-SEM does not impose any strict requirements regarding minimum observations. However, the available social science literature ([79,80]) recommends that a dataset of between 100 to 150 is necessary to obtain a significant model. However, for the PLS-SEM approach, a “10-times rule” is one of the most widely implemented estimation procedures which suggests that the minimum number in the sample should be determined by 10 × multiplication of the highest count of linkages of internal or external indicators pointing to any latent component in the framework [81,82]. In their groundbreaking study on PLS-SEM, F. Hair Jr. et al. [53] provided a substitute for the “10-times approach” for estimating the minimal sample size, popularly known as “the minimum R-squared method”. In the method, three distinct criteria—the maximum number of arrows pointing at a latent variable (construct) in a model, the significance level, and the minimum R^2^ in the model—are applied. The dataset used in this study met the required minimum sample size (Table 2).

## 4. Results

The proposed framework was assessed by employing a reflective indicators model. This concurrently explores the cognitive approaches of the measurement framework and provides an approximation of the model attributes [75]. In line with existing studies (Hair et al. [75], Adnan et al. [84]), a double stage methodology was used for assessing the PLS-SEM outcomes. The first stage examined the measurement framework to ensure the appropriateness, validity, and reliability of the proposed constructs. The second stage involved the exploration of the interrelationships between the latent and observed variables.

### 4.1. Descriptive Statistics

Descriptive statistics are concise explanatory variables that describe a specific information group, which may represent the full population or a sample [85]. Table 3 shows the demographic characteristics of the 499 respondents. Most of the pig breeders were male (71%) and 41% were in the 30-39 age group. Most of the surveyed farmers (41%) had undergone secondary school education, followed by those with no schooling/primary school education (21%), completed a diploma (20%), and those with a bachelor’s degree or above (18%). Around 76% of the respondents had farming experience of 5–10 years, 13% experience of more than 10 years, and 11% experience of 1–5 years. 

### 4.2. Measurement Framework

#### 4.2.1. Construct Reliability

To confirm the internal reliability of the reflective indicators used, a set of reliability tests was employed including assessment of indicator reliability and internal consistency reliability. Internal consistency was determined by comparing the reliability of the observed parameters to a targeted latent construct [86]. In addition, it was necessary to determine if the correlations between the constructs were adequate or not [87]. Bagozzi and Yi [88] and Hulland [89] recommend a value between 0.600–0.700 should be obtained for Cronbach’s alpha and composite reliability. Dijkstra and Henseler, 2015 [90] suggested that, for assessing the Dijstra-Henseler’s rho (rho_A), a cut-off value of 0.70 is acceptable. Table 4 shows that the Cronbach’s alpha values were between 0.819 and 0.968, and the composite reliability scores fluctuated between 0.686 to 0.937, whilst the rho_A values were from 0.702 to 0.978. Based on these findings, it can be concluded that the measurement approach was internally consistent and reliable as the values obtained all satisfied the minimum criteria. To test for any multi-collinearity in the framework, an extra collinearity test was performed, as recommended by Hair et al. [87]; the findings showed that this problem did not occur in the framework.

#### 4.2.2. Convergent Validity

The next assessment involved determining the convergent validity of the indicators. According to Hair et al. [87], it is possible to demonstrate a measure’s convergent validity if it has a strong correlation with other measurements that are used to assess the same concept. It is possible to assess whether the collection of particular elements by which the concept is derived is accurately determined using convergent validity assessment [75]. Ali et al. [91] and Wong [76] suggested that the average variance extracted (AVE) provides the basis for measuring convergent validity. However, Fornell and Larcker [86] and Bagozzi and Yi [88] recommended that an AVE should be 0.500 or more, indicating that at least 50% of the parameter variability is considered. All the items were significantly connected due to their substantial fit; as the AVE values of the study were achieved, the criterion determining convergent validity was met [86].

#### 4.2.3. Discriminant Validity

After confirming convergent validity, discriminant validity was then evaluated. To avoid problems caused by multicollinearity, it is essential for any study that involves latent constructs to evaluate the discriminant validity of the study [92,93]. In psychology, discriminant validity concerns whether constructs or measures that are not possibly related are uncorrelated [94]. According to Campbell and Fiske [95], to establish discriminant validity, it is necessary to show that assessments of conceptually unrelated constructs are not substantially interlinked. In practice, the value of a measure of discriminant validity needs to be lower than the value of a measure of convergent validity [96]. Therefore, discriminant validity measurements were assessed to ensure that each indicator was clearly distinguished within the construct [97]. The study evaluated the discriminant validity using three distinct criteria: (i) the Fornell–Larcker criterion, (ii) cross-loading assessment, and (iii) heterotrait-monorail correlations (HTMT), as recommended by Adnan et al. [84] and Munim and Noor [98]. According to the Fornell–Larcker [86] criteria, researchers may determine how much shared variation there is across the model’s latent constructs. To determine whether the measurement model’s convergent validity satisfies this condition, the average variance extracted (AVE) and composite reliability metrics should be examined (CR), as suggested by Dias et al. [99], Hamid et al. [100] and Wong [76]. According to the Fornell–Larcker criterion [86] and the cross-loadings [101], the constructs’ discriminant validity was established, as shown in Table 5; in particular, (i) it was shown that the square root of the AVE of each structure was greater than the linear relationship it had with other structure, and (ii) each item was weighted most strongly on its structure.

Finally, the study assessed the HTMT criterion developed and popularized by Henseler et al. [102]. The HTMT test is a unique criterion based on the average correlations of the variables within their associated constructs [103]. Table 6 shows that all the indicator HTMT values were less than the threshold value of 0.900, which indicates that the proposed framework possessed an adequate level of discriminant validity, as recommended by Henseler et al. [102]. 

### 4.3. Fitting Outcomes within the Structural Model

After validating the measurement framework, the next step in PLS-SEM is to fit the measurement model within the aspects of the structural model [75,79]. Structural modeling shows if the qualitative and quantitative aspects of the proposed framework are adequately connected [104]. To ensure a cohesive relationship between the internal and external framework, the researchers used bootstrapping strategies involving the production of t-statistics, as recommended by Wong [76]. The technique has been widely used in similar studies (such as Sarkar et al. [104], Wei et al. [105], and Wang [106]). The bootstrapping method generates subsamples from the initial dataset that are comprised of random observations generated by the replacement of subsamples [107]. A replication process was undertaken employing 5000 subsamples from the original sample to generate bootstrap errors. To complete this, the “Calculate” menu in the SmartPLS-3 software was used and the “Bootstrapping” option taken for 499 cases with 5000 samples. Hair et al. [108] suggested that to verify the reliability of the structural model the “t-values” should be greater than 1.96. Table 7 indicates that all the measures possessed acceptable values of latent variables in the correlational matrix. Therefore, it was confirmed that the structured model had been properly constructed and that there was a sufficient level of interaction between the theory and the constructs. It was also concluded that the incentive and disincentive mechanisms were significantly linked with respect to environmentally friendly livestock waste management practice. 

## 5. Conclusions

Waste management is important as it protects the environment from the toxic effects of inorganic and biodegradable elements present in the waste. It would be dangerous for livestock resources, public health, and the ecosystem itself if were not managed effectively. The proper management of manure and biological waste from livestock sectors has become a “buzzword” for governmental bodies, academia, and international organizations. However, in emerging countries, there is a huge gap between the recommended processes for managing waste and the actual behavior of farmers. Therefore, governments usually use several techniques, popularly known as incentive and disincentive mechanisms, to improve the situation. Though the existing study comprehensively explored the extent of incentive and disincentive mechanisms, most studies have explored these two crucial factors in an isolated manner. Moreover, outcomes from these empirical studies were not consistent, and there has been no consensus in terms of theoretical analysis. Therefore, based on the variables extracted from an extensive literature review, a theoretical model was proposed, which can combine these two interrelated mechanisms into an integrated framework, which was tested based on data collected from 499 pig breeders in China. The results indicated a significant interaction between incentive and disincentive mechanisms for ensuring environmentally friendly livestock waste management practices. The survey results showed significant interaction values for the incentive mechanism, while the disincentive mechanism showed relatively lower interaction values. More specifically, discount policy, subsidy policy, and free training were the most important factors impacting the farmers surveyed. In summary, it can be inferred that, if incentive and disincentive mechanisms work well, they can encourage the adoption of environmentally friendly livestock waste management practices by farmers. Therefore, the proposed hypothesis was supported as government incentive and disincentive mechanisms were found to influence farmer’s behavior towards the adoption of environmentally friendly livestock waste management practices.

Based on analysis of the results, the following policy implications are suggested: (i) As the study found that disincentive mechanisms had relatively lower interaction values, the government should extend regulatory and direct penalties and work closely with local authorities to implement disincentive mechanisms in the most effective manner. The government should ensure targeted implementation of disincentive mechanisms, based on quantitative assessment; (ii) Training programs should be arranged more frequently and support increased for bonus community service policy. Subsidy policy should be strengthened based on the local context. In addition, discounts on various technical and mechanical instruments and technologies should be more widely applied; (iii) Government should extend its support for and promote the practical importance and usefulness of environmentally friendly livestock waste management practices. Moreover, the government should work on enhancing the knowledge of farmers and motivating them regarding the benefits of environmentally friendly livestock waste management practices.

There are some limitations of the study. Behavior is dynamic and changes depending on the variables affecting it, however, the interactions among the controlling variables was not fully evaluated in the study. Future studies should consider the impacts of control variables within a core framework using more straightforward models, such as radial, additive, and slack-based measure models. Second, the research used PLS-SEM as the analytic approach. Future studies should consider the use of mixed methods, such as partial least squares structural equation modeling (PLS-SEM) and covariance-based SEM (CB-SEM), to produce more robust outcomes. The article did not explore the impacts of socio-economic factors and previous behavioral commitments; therefore, future studies should extend the proposed framework to incorporate socio-economic dynamics and prior cognitive factors. Finally, the measurement of farmers’ commitment and personal beliefs is useful to enhance understanding of farmer’s behavior. Future studies should employ pertinent psychological theories, such as the theory of planned behavior (TPB), game theory, and the theory of protection motivation, to construct comprehensive explanatory frameworks.

## Figures and Tables

**Table 1:** Selected variables.

Variable		Description	Likert Scale
Disincentive mechanism	Regulatory policy	Does the regulatory policy affect your family’s behavior toward environmentally friendly livestock waste management?	Ranges from 1–7 (Strongly agree, Agree, Slightly agree, Neutral, Slightly Disagree, Disagree, Strongly disagree)
	Penalty policy	Does the monetary and social penalty policy affect your family’s behavior for environmentally friendly livestock waste management?	
	Disincentive policy	Does the disincentive policy of cracking down on the trading in the underground market affect your family’s behavior for environmentally friendly livestock waste management?	
Incentive Mechanism	Subsidy policy	Does the subsidy policy impact your family’s behavior toward environmentally friendly livestock waste management?	
	Insurance policy	Does the policy linking insurance impact the environmentally friendly livestock waste management behavior in your family?	
	Bonus community service policy	Does the bonus community service policy have any impact on your family’s behavior for environmentally friendly livestock waste management?	
	Discount policy	Does the loan interest discount policy affect the family’s behavior regarding environmentally friendly livestock waste management?	
	Free training	Does free technical training affect your family’s behavior regarding environmentally friendly livestock waste management?	

**Table 2:** Minimum R-squared method for minimum sample size estimation (Adopted from Kock and Hadaya [83]).

Maximum Number of Arrows Pointing at a Construct	Minimum R^2^ in the Model			
	0.10	0.25	0.50	0.75
2	110	52	33	26
3	124	59	38	30
4	137	65	42	33
5	147	70	45	36
6	157	75	48	39
7	166	80	51	41
8	174	84	54	44
9	181	88	57	46
10	189	91	59	48

**Table 3:** Demographic characteristics of the respondents.

Characteristics	Classifications	Frequency	Percentage (%)
Gender	Male	356	71%
	Female	143	29%
Age	18–29	98	20%
	30–39	204	41%
	40–49	135	27%
	50 and above	62	12%
Education level	No Schooling/Primary School	106	21%
	Secondary School	207	41%
	Diploma	97	20%
	Bachelor’s Degree and above	89	18%
Marital status	Single	104	21%
	Married	309	62%
	Divorced	34	7%
	Widow/Widower	52	10%
Working Experience as a farmer	1–5	58	11%
	5–10	378	76%
	More than 10 years	63	13%

**Table 4:** Validity of constructs (reflective outer models).

Construct	Code	α	Indicator Reliability	rho_A	VIF	CR	AVE
Regulatory policy	RP_	0.828	0.686	0.702	1.000	0.883	0.754
Penalty policy	PP_	0.880	0.774	0.896	1.654		
Disincentive policy	PUP_	0.895	0.801	0.832	1.456		
Subsidy policy	SP_	0.937	0.878	0.950	1.079	0.942	0.836
Insurance policy	IP_	0.819	0.670	0.827	1.486		
Bonus community service policy	BCSP_	0.883	0.780	0.888	1.079		
Discount policy	DP_	0.968	0.937	0.978	1.600		
Free training	FT_	0.956	0.914	0.968	1.516		

Note: α = Cronbach’s Alpha; rho_A = Dijstra-Henseler’s rho; CR = Composite reliability; AVE = Average variance extracted; VIF = Variance inflation factor.

**Table 5:** Fornell–Larcker criterion for measuring discriminant validity.

	1	2	3	4	5	6	7	8
Regulatory Policy	**0.828**							
Penalty Policy	0.379	**0.880**						
Disincentive Policy	0.644	0.357	**0.895**					
Subsidy Policy	0.584	0.156	0.456	**0.937**				
Insurance Policy	0.246	0.379	0.468	0.544	**0.819**			
Bonus Community Service Policy	0.368	0.135	0.544	0.478	0.478	**0.884**		
Discount Policy	0.433	0.189	0.456	0.268	0.376	0.544	**0.968**	
Free Training	0.532	0.135	0.245	0.457	0.368	0.353	0.136	**0.956**

**Table 6:** HTMT criterion.

	1	2	3	4	5	6	7
Regulatory Policy	**-**						
Penalty Policy	**0.453**						
Disincentive Policy	0.223	**0.686**					
Subsidy Policy	0.128	0.338	**0.557**				
Insurance Policy	0.332	0.146	0.468	**0.797**			
Bonus Community Service Policy	0.234	0.464	0.544	0.478	**0.702**		
Discount Policy	0.144	0.209	0.456	0.268	0.250	**0.570**	
Free Training	0.032	0.176	0.386	0.153	0.287	0.269	**0.468**

**Table 7:** Bootstrap results of the model (inner model).

Hypothesis	Total Sample Estimate	Mean of Subsample	Standard Error	t-Statistics	Outcomes
Disincentive mechanism—Ensuring environmentally friendly livestock waste management	0.764	0.698	0.089	9.16	Supported
Incentive Mechanism—Ensuring environmentally friendly livestock waste management	0.909	0.823	0.051	19.96	Supported

## Data Availability

The data will be provided upon request by the corresponding author.
